# Result of reclamation of man-made dumps from phosphorite deposits in the semi-desert zone of Kazakhstan

**DOI:** 10.1371/journal.pone.0317500

**Published:** 2025-02-24

**Authors:** Aizhan Konysbayeva, Zura Yessimsiitova, Murat Toktar, Alibek Mutushev, Yryszhan Zhakypbek, Serik Tursbekov, Galiya Tursbekova, Zhenis Kozhayev, Assel Kozhamzharova, Serzhan Mombekov, Saki Raheem

**Affiliations:** 1 Al-Farabi Kazakh National University, Almaty, Kazakhstan; 2 Kazakh Research Institute of Soil Science and Agricultural Chemistry Named After U.Uspanov, Almaty, Kazakhstan; 3 LLC «Scientific production technical center «Jalyn»», Almaty, Kazakhstan; 4 Institute Mining and Metallurgical Institute named after O.A. Baikonurov, Satbayev University, Almaty, Kazakhstan; 5 International Engineering-Technological University Kazakhstan, Almaty, Kazakhstan; 6 School of Pharmacy, JSC “S.D. Asfendiyarov Kazakh National Medical University”, Almaty, Kazakhstan; 7 School of Life Sciences, University of Westminster, London, United Kingdom; Veracruzana University: Universidad Veracruzana, MEXICO

## Abstract

Pollution from industrial activities, including heavy metal contamination, poses severe environmental challenges, especially in industrialized regions. This study evaluates reclamation efforts at the Kokzhon phosphorite deposit in Kazakhstan’s semi-desert zone, where over 67 million tons of industrial waste have accumulated across 3.3 thousand hectares. Reclamation efforts encompassed the treatment of 6,400 hectares using carbamide amendments and the planting of resilient phytomeliorants, including Russian Olive, Black Saxaul, Androsov Elm, and Salt Cedar. While tree survival rates were low (11%), herbaceous vegetation achieved remarkable success, with legumes and cereals attaining 95% growth rates. Herbaceous productivity increased from 2,200 kg/ha in 2013 to 3,300 kg/ha in 2018, alongside vegetation cover expanding from 60% to 80%. Soil fertility also improved significantly, with humus content rising from 0.18% in 2012 to 1.14% in 2023. Despite these improvements, the long-term impacts of industrial phosphorite mining continue to challenge ecosystem recovery. Over a 12-year period, reductions in humus content (47.6%) and herbaceous productivity (28.4%) have been observed, highlighting the need for enhanced soil management strategies to sustain reclamation outcomes. The results emphasize the potential of biological reclamation to restore degraded semi-desert ecosystems while underscoring the necessity of scalable, cost-effective solutions and long-term monitoring to mitigate ongoing environmental damage.

## Introduction

Growing industrial activities have significantly transformed natural systems, surpassing many natural processes in scale and impact. The intensive extraction of oil, ore, coal, and gas containing radionuclides, along with the development of nuclear energy, has dramatically increased the presence of both natural and artificial radionuclides and heavy metals in the environment [1,2]. Abandoned mining and processing enterprises, characterized by high levels of radioactivity, have further contributed to environmental pollution [3]. These anthropogenic impacts are evident both regionally and globally, with increasing human pressure resulting in zones of critical environmental challenges, particularly in Kazakhstan.

Kazakhstan faces numerous challenges in managing water resources within the rapidly expanding mining sector. These challenges stem from the country’s arid climate, water scarcity, and growing industrial demands, particularly from mining operations vital to the national economy [4]. Limited freshwater resources and inadequate hydrological data hinder assessments of water trends and the development of effective management strategies [4,5]. As a significant consumer of water, the mining industry intensifies competition with agriculture and other sectors, raising concerns about sustainability. This over-reliance on scarce water resources underscores the urgent need for improved water-use efficiency and management practices [4].

Despite some mining operations adopting water recycling practices, inefficiencies remain. Excessive water consumption and significant losses during mining processes highlight the urgent need for improved water-use efficiency across the sector. Additionally, Kazakhstan’s regulations and institutional frameworks for mine water management are underdeveloped and poorly enforced. The lack of standardized water accounting practices hampers the ability of government and industry to effectively mitigate environmental risks. Insufficient regulatory oversight further intensifies challenges related to unsustainable water use [4].

Mining activities generate significant waste, adversely impacting ecosystems and landscapes. These wastes pose risks to both the environment and human health, particularly through vegetation damage [6,7]. The surface layer of soil, essential for restoring areas damaged by mining, is often severely degraded. This degradation complicates ecosystem restoration due to the soil’s poor physical and chemical properties [8]. Anthropogenic activities, such as mining, disrupt soil structure, impairing its ability to facilitate air and water exchange [9].

Reclamation of damaged lands involves restoring soil fertility and creating favorable conditions sustainable use [10–12]. At the Kokzhon site, reclamation focused on addressing the low organic matter content of the soil and its unique composition of dolomite, quartz, and slate rocks. These materials form the foundation of the reclaimed soil, which are otherwise classified as industrial waste. The low concentration of heavy metals, except for slightly elevated cadmium (Cd) levels, enabled a focus on stabilizing soil and improving fertility through vegetation [13].

This process often focuses on improving soil properties and enhancing microclimatic conditions, which are often unfavorable for plant growth [14]. Consequently, reclamation efforts are directly influenced by environmental conditions. For instance, the Kokzhon phosphorite deposits, located in a semi-desert zone, are subject to adverse climatic factors that significantly affect biological reclamation [13].

Restoring the vegetation cover in man-made, damaged areas of semi-desert regions presents considerable challenges [15]. The extinction of plant communities naturally adapted to those regions means that degraded desert and semi-desert ecosystems take a long time to recover, posing strategic management difficulties [16]. Soil nutrient composition and structure are also significantly affected during this process [17]. Transition zones between desert and semi-desert regions are highly sensitive to environmental harm. Thus, vegetation restoration and the creation of botanical gardens are effective methods to improve surface areas affected by dumps [18,19].

Vegetation plays a vital role in stabilizing soil by accumulating small particles and protecting the soil surface from erosion. Root systems help prevent soil degradation and maintain stability. Furthermore, restored vegetation increases the organic matter content of soils, improving its quality [20,21].

In the restoration of eroded ecosystems, the use of cultivated plants species resistant to dump materials and adverse conditions can effectively stabilize soil structure on dump surfaces [22,23]. Fast-growing, desert-resistant plant species can thrive on soils with low nutrient content when carefully selected. These plants must exhibit robust systems capable of rapid recover and distribution [24,25]. After reclamation, vegetation cover protects the soil from erosion, counteracts land degradation and desertification, and significantly enhances agricultural and livestock productivity [26–28].

At the Kokzhon phosphorite deposits, which severely damage soil cover and vegetation, it is crucial to rehabilitate technologically damaged agrolands through the use of phytomeliorants and the establishment of biological communities. This study evaluates the effectiveness of biological reclamation techniques using locally adapted phytomeliorants to address the challenges posed by industrially degraded soils. The research focuses on improving soil fertility, stabilizing loose soil, and enhancing vegetation cover using species resilient to arid and semi-arid conditions. By integrating innovative soil amendments and long-term vegetation monitoring, this study contributes to the growing body of knowledge on sustainable land reclamation in semi-desert regions. The findings are expected to inform future reclamation strategies and provide a replicable framework for restoring industrially damaged landscapes in similar ecological contexts.

## Materials and methods

### Ethics Statement

Ethical approval was obtained from Kazphosphate Corporation, and informed written consent was obtained prior to conducting the study.

### Inclusivity in global research

Additional information regarding the ethical, cultural, and scientific considerations specific to inclusivity in global research is included in the Supporting Information (S1 File).

### Study area

**Fig 1:**
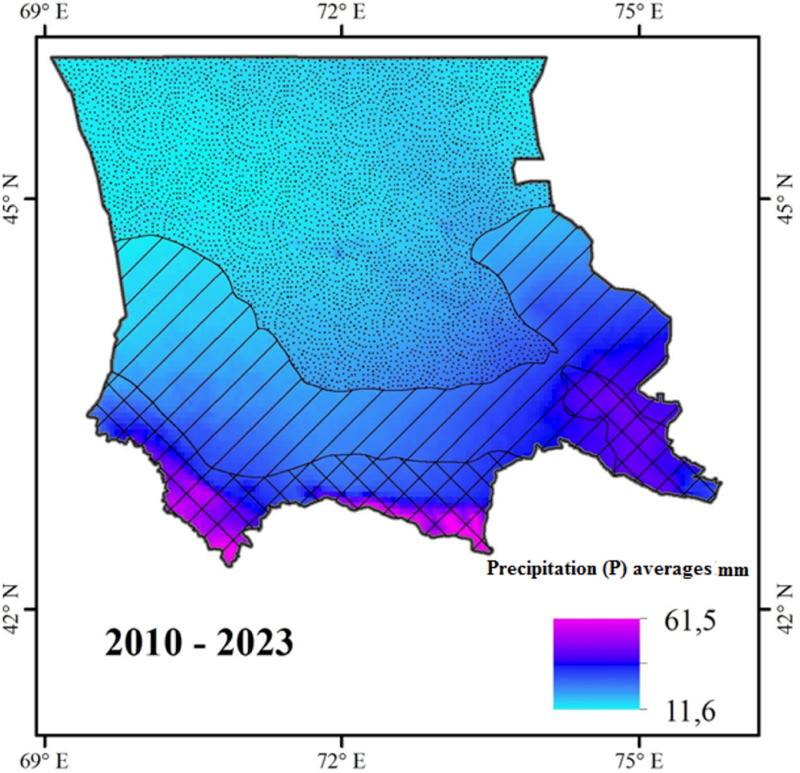
Average monthly precipitation for the Zhambyl region over a 10-year period (2010 – 2023).

The study focuses on the Kokzhon phosphorite deposit located in the Zhambyl region of Kazakhstan (43°33’16.72” N; 69°31’50.09” E). The total area of the Kokzhon deposit is approximately 300 hectares. The region is characterized by a dry climate, with annual rainfall ranging between 200 and 250 mm. Climate data, including temperature (T) and precipitation (P) averages, were analyzed for the period from 2010 to 2023 (Fig 1) using TerraClimate data. Monthly climate and water balance data for global terrestrial surfaces were provided by the University of Idaho. Data analysis was conducted using the Google Earth Engine could platform, and the resulting map was exported to ArcGIS 10.8 for layout and final visualization.

The average annual temperature in the region ranges from 6.5 °C - 10.5 °C, with mountainous and northern areas averaging 6.5 °C to 8 °C, and central areas averaging 9 °C to 10 °C. During warmer periods, temperature average 15 °C to 17 °C in mountainous areas and 18 °C to 19 °C in the central areas. Extreme temperatures can reach 45 °C to 47 °C in desert zones and 40 °C to 42 °C in mountainous regions.

**Fig 2:**
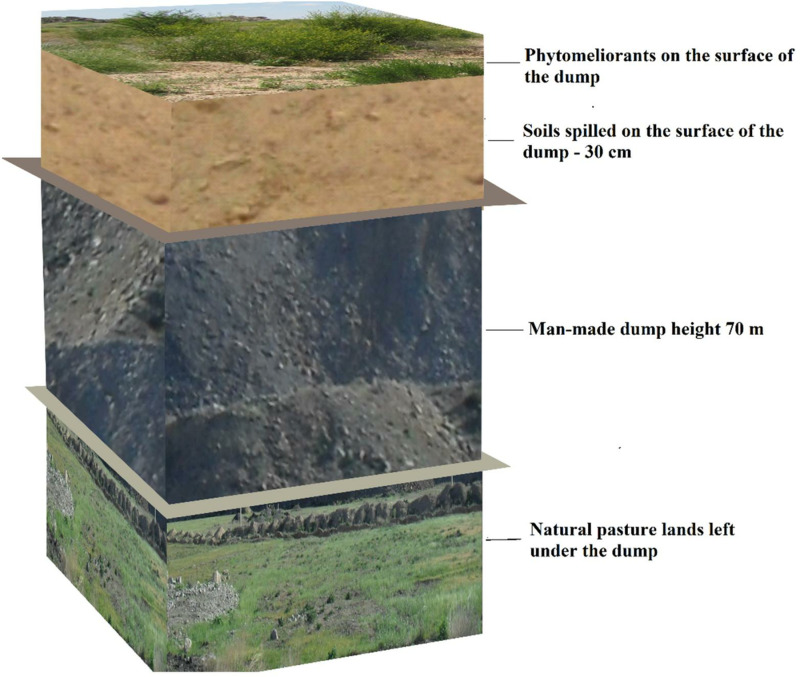
General reclamation scheme of the heap.

An experimental plot measuring 110 m ×  115 m was selected for the study. Reclamation work was carried out in two stages: technical and biological. During the technical stage, the surface of the dump was leveled, and 700 tons of soil were added to create a 30 cm thick layer (Fig 2).

**Fig 3:**
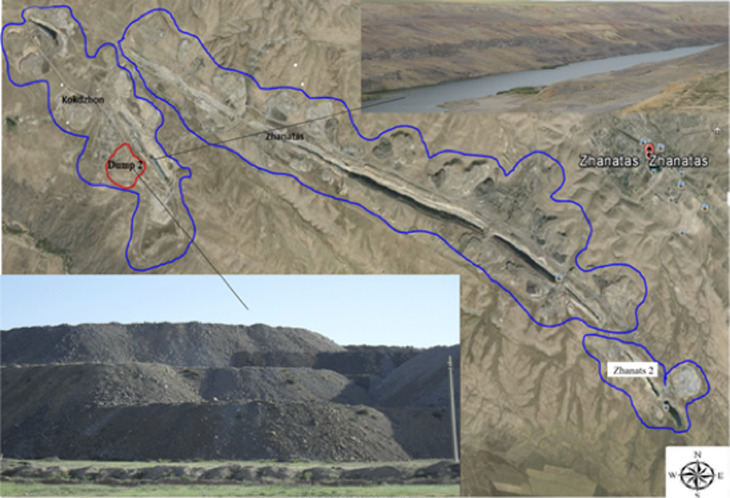
Study area and sampling points.

The Kokzhon phosphorite deposits are located at an altitude of 500 to 700 meters above sea level. The site consists of multi-storey industrial dumps and several large quarries, ranging from 1.6 to 2.98 km in length, 360 to 430 m in width, and 90 to 95 m in depth (Fig 3). There are three industrial dumps, each with a height of 50 to 70 m, covering a total area of 16 to 27 hectares [29,30]. Overall, the Kokzhon phosphorite spans more than 1000 hectares.

### Materials and research methods

In October 2012, technical reclamation of the surface of the Kokzhon dump was conducted, followed by dynamic monitoring of the planted phytomeliorants on the man-made dump.

The technical reclamation phase involved clearing large boulders from the dump’s surface and filling pits. The surface was then leveled with mixed rocks to prepare it for vegetation. Additionally, 700 tons of sand, forming a 30 cm thick layer, were spread and leveled to meet technical requirements [13].

**Fig 4:**
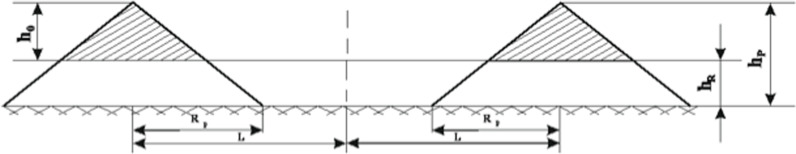
Diagram of soil dumping in technical reclamation on a dump.


Soil aggregates were distributed systematically across the dump’s surface to meet specific reclamation needs. The required volume of soil was calculated based on the vehicle’s body capacity, the surface area of the reclamation dump, and the thickness of the soil layer. The reclamation area was divided into quadrants measuring 2 L (Fig 4) where L was calculated using formula (1).

$$L=0.5\sqrt{\frac{v}{h}}$$ (1)

where:

v is the volume of the dump truck’s body (m^3)^

h is the thickness of the spilled soil layer (m)

**Fig 5:**
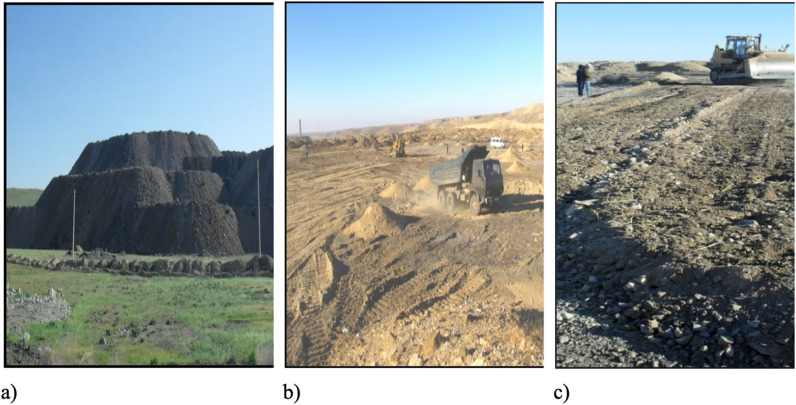
a) dump; b) stage of dumping soil on the mound; c) stage of leveling spilled soil.

At the vertices of these squares, dump trucks discharged the soil aggregates to ensure even material distribution, facilitating efficient leveling for reclamation. Consequently, the spilled soil on the pile’s surface was effectively leveled using a bulldozer (Fig 5a, 5b and 5c).

The volume of soil required for reclamation work was calculated using the following formula (2).


$$V_{p}=S_{P}*h_{H}$$ (2)

Where:

V_P_ = volume of soil required for reclamation works (m^3^).

Sp =  total reclamation area of the dump (m^2^).

h_H_ =  thickness of the soil layer applied to the surface.

After the technical reclamation phase, biological reclamation commenced in late April 2013. In the test area, 600 trees representing four species were planted as part of this reclamation effort. The species selected—Russian Olive (*Elaeagnus angustifolia* L.), Black Saxaul (*Haloxylon aphyllum* (Minkw.) Iljin), Androsov Elm (*Ulmus minor* Mill), and Salt Cedar (*Halimodendron halodendron* (Pall) Voss)—are well-documented for their resilience in arid and semi-arid environments [13]. These species thrive in poor, saline soils and drought conditions, making them ideal candidates for restoring the Kokzhon dumps. The 600 trees were evenly distributed among the four species (150 per species). Biochar (150 g) and urea (70 g) were added to each planting hole (40 cm ×  40 cm). Additionally, herbal phytomeliorants were applied at a rate of 10 tons per hectare and incorporated into the top 30 cm of soil.

Air-dried soil samples were gently crushed and passed through a 2 mm sieve. Soil analyses were conducted in accordance with the guidelines outlined in the Russian Handbook on the Chemical Analysis of Soils [31]. Soil particle distribution was determined using Kachinsky’s method [32], total carbonates via the gas-volumetric method [33], total potassium through the Michigan method [34], and heavy metals (Zn, Cu, Pb, Cd) were analyzed using elementary analysis [35]. The biological mass of plants was assessed using four replicates per square meter. Statistical analyses, including Fisher’s Exact Test, were conducted to compare survival rates among species and assess differences in their adaptation and resilience. Confidence intervals (95% CI) validated the results, capturing the observed variability in survival rates across species.

## Results and discussion

During the technical reclamation period, heavy metals were detected in the spilled soils. The concentrations of heavy metals in the topsoil (Table 1) were below the proposed thresholds for European soils [36], with the exception of Cd. The sub-alkaline oil pH (~8.4) played a beneficial role in reducing the solubility and mobility of heavy metals. Higher pH levels stabilize heavy metals, limiting their bioavailability and mitigating environmental impact. This observation aligns with findings by Toktar et al. [13], who emphasized that alkaline conditions in reclaimed semi-desert regions significantly limit heavy metals migration and enhance soil stabilization. The stabilization of Pb, Zn, and Cu observed in this study is consistent with similar findings from other reclaimed sites, where alkaline conditions inhibited heavy metal leaching into groundwater systems [13,37].

**Table 1:** Heavy metals in the topsoil (0-30 cm) of the test area (N =  23), 2012.

Metal	Form	Mean mg kg^-1^	Min	Max	Max-Min	SD	Threshold*
Cd	total	3.4	3.2	3.6	0.4	0.23	1.5
	movable	1	0.9	1.2	0.3	0.2	
Cu	total	26.8	25	28	3	1.53	100
	movable	2.9	2.1	3.7	1.6	0.7	
Pb	total	34.1	29	39.6	10.6	4.7	100
	movable	13.6	11	15.6	4.6	2.2	
Zn	total	58.5	54	62.4	8.4	3.7	200
	movable	2.2	1.2	2.8	1.6	0.72	

*Proposed threshold values with soil pH >  7 [36].

However, the elevated Cd concentration (3.4 mg/kg) slightly exceeded the proposed thresholds, emphasizing the need for continuous monitoring in reclaimed areas. The mobility of Cd is less influenced by alkaline pH compared to other metals. Studies by Sheoran et al. [37] have demonstrated that organic amendments, such as biochar, can further reduce Cd availability by enhancing adsorption and complexation within the soil matrix.

The findings indicate that the dump, composed of underground rocks from dry residual materials, lacks significant concentrations of heavy metals harmful to soil and plants. These results are consistent with Tokar et al. [13,30], who observed that high pH and low water activity in arid reclaimed soils contribute to heavy metal stabilization. Together, these factors reduce the risk of metal uptake by plants and leaching into the surrounding environment.

Laboratory analyses of soil samples from the 0-30 cm sandstone layer during the technical reclamation phase revealed a granulometric composition dominated by coarse, dusty, sandy fractions. Sandy fractions accounted for 38.4%, silt fractions for 51.72%, and clay fractions for 13.4%. This composition is unfavorable for plant growth, as it limits water retention and hinders the formation of stable soil aggregates. Toktar et al. [16,30] similarly noted that high silt and sandy fractions in reclaimed soils pose challenges for water-holding capacity and nutrient retention, particularly in semi-arid climates.

The humus content in the reclaimed soil was very low (0.18%), emphasizing the challenges of restoring organic matter in degraded soils. Similarly, the average levels of total nitrogen (0.035%), phosphorus (0.08%), and potassium (0.56%) were suboptimal. These findings align with studies from other semi-arid mining regions, where poor nutrient profiles limit plant establishment and growth [37]. These deficiencies highlight the importance of integrating organic amendments and herbaceous phytomeliorants to accelerate the recovery of soil fertility and structure.

The total exchange absorption complex was measured at 9.2 mg/kg, and the soil exhibited alkaline properties, with a pH value of 8.41 (Table 2). This alkalinity is typical of regional reclamation sites, where naturally alkaline conditions favor the immobilization of heavy metals. However, additional amendments are often required to improve cation exchange capacity and enhance nutrient availability [38].

**Table 2:** Parameters of soil grunts on experimental sites (N =  23) 2012.

Parameters	Mean	Min	Max	Мах- Min.	SD
Sand (1.0–0.25 mm)	3.81	2.25	4.72	2.47	1.16
Sand (0.25–0.05 mm) %	34.6	32.5	36.5	4	1.64
Silt (0.05–0.01 mm) %	32.4	30.8	33.2	2.4	1.1
Silt (0.01–0.005 mm) %	10.3	7.3	12.6	5.3	1.1
Silt (0.005–0.001 mm) %	9.22	4.47	16.2	11.7	4.96
Clay (<0.001 mm) %	13.4	11.3	16.2	4.9	2.1
Humus (%)	0.18	0.16	0.2	0.04	0.02
Gypsum (%)	0.15	0.07	0.26	0.19	0.08
N tot (%)	0.035	0.03	0.04	0.01	0.008
N hydrolyzable (mg kg^ − 1^)	9.1	5.6	14	8.4	3.52
CaCO_3_ (%)	3.52	2.6	4	1.4	0.54
P_2_O_5_ total (%)	0.08	0.04	0.12	0.08	0.03
P_2_O_5_ movable (mg kg^ − 1^)	4.75	1	12	11	2.59
K_2_O total (%)	0.56	0.17	0.9	0.73	0.31
K_2_O movable (mg kg^ − 1^)	148.4	141.2	155.4	14.2	5.86
Reaction (pH)	8.41	8.34	8.52	0.18	0.08
Caexc (cmol + kg^ − 1^)	5.1	4.67	5.17	0.5	0.25
Mgexc (cmol + kg^ − 1^)	2.99	2.1	3.69	1.59	0.64
Naexc (cmol+kg^ − 1^)	0.95	0.73	1.15	0.42	0.19
Kexc (cmol + kg^ − 1^)	0.15	0.11	0.18	0.07	0.04

In the 2013 biological reclamation experiment, soil samples from the 0-30 cm layer revealed a granulometric composition of 27.3% sandy fractions, 54.4% silt fractions, and 18.3% clay fractions. This shift in granulometric composition compared to 2012 suggests the leaching of finer fractions into lower layers. Such patterns are consistent with findings from other arid-region reclamation studies, where soil aggregation processes are influenced by water infiltration rates and the use of organic amendments [13]. However, the predominance of dust fractions continued to pose challenges for plant growth and soil aggregate formation. Similar observations were reported by Adenova et al. [40], who noted that finer fractions in semi-desert soils limit water retention and slow soil aggregation despite external interventions.

Agrochemical analysis indicated slight improvements in fertility parameters compared to 2012. Humus content increased to 0.35%, total nitrogen to 0.042%, total phosphorus to 0.09%, and total posassium to 0.7%. These results align with Kabir et al. [40] who reported marginal but progressive improvements in organic carbon and nutrient retention in biochar-amended reclamation plots in semi-arid zones. While these amendments enhance soil fertility, the persistence of an alkaline pH (8.4) underscores the need for additional soil conditioning measures to optimize nutrient availability and microbial activity, particularly in alkaline soils.

In 2023, a comparative analysis of the 2013 soil data was conducted as part of ongoing monitoring of the experimental plots. Over the 10-year period, a significant improvement in soil fertility was observed. One notable change was a 3.3-fold increase in humus content, rising from 0.35% in 2013 to 1.14% in 2023. This improvement can be attributed to the decomposition of biochar and the gradual accumulation of organic matter from herbaceous phytomeliorants. These results are consistent with findings from studies on the long-term effects of organic amendments, which highlight biochar’s role in stabilizing organic carbon and promoting microbial colonization as critical factors for enhancing soil fertility, especially in semi-desert reclamation efforts [39,41].

The application of biochar and carbamide significantly improved soil properties and vegetation growth over time. Enhanced nutrient availability and moisture retention contributed to better plant survival and growth rates across the reclaimed areas. However, the study did not directly compare biochar and carbamide with alternative soil amendments, such as compost or green manure, which could potentially offer similar benefits at lower costs. The scalability of biochar application remains constrained by the high costs of large-scale use and the labor required for consistent application. Future reclamation efforts could explore combininig biochar with compost or green manure to leverage complementary mechanisms of nutrient release and soil improvement.

Despite these limitations, the long-term benefits of biochar are evident. Its ability to stabilize nutrients, enhance microbial activity, and retain moisture makes it an effective amendment for reclamation in semi-arid environments. However, consistent monitoring is essential to ensure sustained soil health and fertility [41].

To maintain logical balance and prevent unintended consequences, careful attention was given to the potential invasiveness of the selected species. None of the chosen species are classified as invasive in the region. Studies conducted prior to their introduction confirmed their ability to coexist with local flora without causing ecological imbalance [42,43]. This aligns with the broader reclamation objective of integrating sustainable vegetation while mitigating the risks associated with introducing non-native, invasive species.

Changes in soil texture were also observed, with an increase in silt and clay fractions. Notably, clay content increased from 18.3% in 2013 to 23.3% in 2023, suggesting the formation of new soil structures due to organic matter decomposition. This trend aligns with findings by Toktar et al. [13], who observed enhanced fine-particle binding in reclamation plots treated with biochar and phytomeliorants. However, the decline in total and mobile potassium levels highlights potential nutrient leaching challenges, consistent with findings from other arid regions, where potassium is prone to deep soil percolation under high infiltration rates [39].

**Table 3:** Laboratory analysis of the physico-chemical properties of soils samples (0 - 30 cm) from 2013 and 2023 (N =  23).

Parameters	Mean	Min	Max	Мах- Min	SD
2013					
Sand (1.0–0.25 mm)	5.4	2.93	14.8	11.87	2.4
Sand (0.25–0.05 mm) %	21.9	15.1	32.1	17.0	4.5
Silt (0.05–0.01 mm) %	29.5	21.7	50.5	45.8	6.4
Silt (0.01–0.005 mm) %	8.6	4.6	16.8	12.2	2.3
Silt (0.005–0.001 mm) %	16.3	2.5	20.6	18.1	3.8
Clay (<0.001 mm) %	18.3	0.5	23.1	40.7	4.5
Humus (%)	0.35	0.07	0.52	0.45	0.14
Gypsum (%)	1.9	0.2	4.6	4.4	1.2
N tot (%)	0.042	0.014	0.07	0.056	0.02
N hydrolyzable (mg kg^ − 1^)	28.5	19.6	36.4	16.8	5.3
CaCO_3_ (%)	3.64	2.71	4.3	1.21	0.53
P_2_O_5_ total (%)	0.09	0.05	0.14	0.09	0.03
P_2_O_5_ movable (mg kg^ − 1^)	5	4	9	5	1.3
K_2_O total (%)	0.7	0.2	0.94	0.92	0.2
K_2_O movable (mg kg^ − 1^)	87	60	150	90	32.4
Reaction (pH)	8.4	8.34	8.52	0.18	0.07
Caexc (cmol + kg^ − 1^)	11	8	13	5	1.1
Mgexc (cmol + kg^ − 1^)	3.9	2.8	5	2.2	0.6
Naexc (cmol+kg^ − 1^)	1.14	0.77	1.66	0.89	0.21
Kexc (cmol + kg^ − 1^)	0.08	0.05	0.16	0.11	0.03
2023					
Sand (1.0–0.25 mm)	7.1	6.7	7.5	0.8	0.4
Sand (0.25–0.05 mm) %	20.7	19.8	21.5	1.7	0.85
Silt (0.05–0.01 mm) %	26.8	25.9	27.9	2	1.1
Silt (0.01–0.005 mm) %	7.9	7.6	8.2	0.6	0.31
Silt (0.005–0.001 mm) %	14.2	13.4	14.7	1.3	0.72
Clay (<0.001 mm) %	23.3	22.9	23.8	0.9	0.45
Humus (%)	1.14	1.1	1.23	0.13	0.07
Gypsum (%)	1.92	1.91	1.93	0.02	0.01
N tot (%)	0.057	0.056	0.059	0.003	0.001
N hydrolyzable (mg kg^ − 1^)	32.4	31.5	32.9	1.4	0.78
CaCO_3_ (%)	3.67	3.49	3,97	0.48	0.26
P_2_O_5_ total (%)	0.11	0.1	0.14	0.04	0.02
P_2_O_5_ movable (mg kg^ − 1^)	5.4	5.1	5.7	0.6	0.31
K_2_O total (%)	0.61	0.54	0.67	0.13	0.07
K_2_O movable (mg kg^ − 1^)	79.5	79.7	80.3	0.6	0.97
Reaction (pH)	8.34	8.31	8.36	0.05	0.03
Caexc (cmol + kg^ − 1^)	11.6	11.2	11.8	0.6	0.32
Mgexc (cmol + kg^ − 1^)	4.1	3.9	4,4	0.5	0.25
Naexc (cmol+kg^ − 1^)	1.15	1.14	1.16	0.02	0.01
Kexc (cmol + kg^ − 1^)	0.075	0.068	0.084	0.02	0.008

Plant growth monitoring revealed steady adaptation, with herbaceous plants such as alfalfa, sainfoin, and grasses demonstrating robust growth. By 2018, these plants had significantly spread beyond the initial sowing plots, reflecting improved soil conditions and the facilitation of natural seed drift, as also observed by Toktar et al. [13]. The dynamic growth rates of tree-shrub plants improved from 4.1% in 2014 to 11% in 2015, underscoring the importance of biochar in supporting vegetation establishment in degraded landscapes. These findings align with observations by Adenova et al. [39], who highlighted the synergistic effects of organic amendments and phytomeliorants enhancing plant survival and biomass production in reclaimed sites.

By 2023, woody-shrub plant growth remained stable, while herbaceous plants achieved 85% vegetation cover, marking substantial expansion in areas with deposited soils while avoiding rocky sections. Detailed metrics, including changes in humus content and productivity levels of herbaceous and woody-shrub species, are summarized in Tables 4–6. These tables also provide vegetation cover measurements derived using the point quadrat method, ensuring consistent and quantitative assessments.

**Table 4:** Humus composition of the man-made dump and bioproductivity of phytomeliorants [44].

Year	Dump Characteristics	Humus Content, %	Biological productivity of Sowing Herbs, (kg/ha)	Biological Productivity of Tree and Shrub Species
2011	The remains of underground rocks formed from dalamide, quartz, and slate stones 30 years ago. During the initial soil formation, vegetation volume was 5%. Humus layer thickness: 0.2 - 0.5 cm.	0.36	190	–
2012	Soil with a 0 - 30 cm layer added; 2 ha of the area technically reclaimed.	0.18	Technical reclamation stage	Technical reclamation stage
2013	Herbal phytomeliorants introduced at a rate of 10 t/ha of biochar into 0 - 30 cm layer of sown soil.	0.33	2200	Growth 4%, 31 plants rooted.
2014	Growth of herbaceous plants and seed distribution observed. Vegetation cover on embankment: 60%.	0.44	2900	Growth 11%, 66 bushes
2018	Increase herbceous plants observed on the embankment and seed drift to other areas. Vegetation cover: 80%.	1.1	3300	Growth 11%, 66 bushes

**Table 5:** Humus content in zonal soils and biological productivity of plants [44].

Year	Depth, (cm)	Characterization of Zonal Soil	Humus Content, %	Biological Productivity of Herbaceous Plants, (kg/ha)	Biological Productivity of Tree and Shrub Species
2011	0 - 10	Located 4 km from the dump. The vegetation cover is 80%.	1.2	2400	–
2018	0 - 10	Located 4 km from the dump. Vegetation cover is 70 - 75%.	0.77	2000	–

**Table 6.** Humus content in zonal soils and dum biological productivity of plants 2023.

Year	Depth, (cm)	Characterization of Zonal Soil	Humus Content, %	Biological Productivity of Herbaceous Plants, (kg/ha)	Biological Productivity of Tree and Shrub Species
2023	0 - 10	Located 1 km from the dump. Vegetation cover is 65 - 70%.	0.70	1900	–
2023	0 - 10	On the embankment, an increase in herbal plants was observed; seeds drift occurred in other territories. Vegetation cover is 85 - 90%.	1,14	3350	Growth 11%, 66 bushes

The dynamics of growth and survival among four tree species – Russian Olive, Black Saxaul, Androsov Elm, and Salt Cedar – over a ten-year period illustrate the progress of reclamation efforts. Table 7 provides a longitudinal view of the survival and growth data for these species during 2013, 2018, and 2023.

**Table 7:** Dynamics of Average Growth of Phytomeliorants in the Dump (2013-2023).

Tree species	Number of grafted seedlings	number of sprouted trees	Height, cm	
min	max
2013				
Russian Olive	150	2	55	100
Black Saxaul	150	9	55	90
Androsov Elm	150	8	58	150
Salt Cedar	150	8	30	95
2018				
Russian Olive	150	4	110	185
Black Saxaul	150	13	75	100
Androsov Elm	150	27	120	190
Salt Cedar	150	22	65	110
2023				
Russian Olive	150	4	145	220
Black Saxaul	150	13	100	110
Androsov Elm	150	27	170	230
Salt Cedar	150	22	80	140

In 2013, during the establishment phase, survival rates were relatively low across all species due to the challenging initial conditions. For instance, Russian Olive exhibited only two sprouted trees out of 150, resulting in a survival rate of 1.3%. Androsov Elm and Salt Cedar demonstrated better resillence, with eight sprouted trees each, reflecting the adaptation challenges faced by all species during the initial years of reclamation.

By 2018, during the mid-term adaptation phase, the number of sprouted trees increased significantly for Androsov Elm and Salt Cedar, with 27 and 22 sprouted trees, respectively. Russian Olive and Black Saxaul also showed modest improvement, with 4 and 13 sprouted trees, respectively. Heigh measurements indicated steady growth, with Androsov Elm reaching up to 190 cm and Salt Cedar reaching 110 cm. These results underscore the positive impacts of reclamation efforts, particularly the use of biochar and carbamide, in improving soil fertility and plant growth.

**Fig 6:**
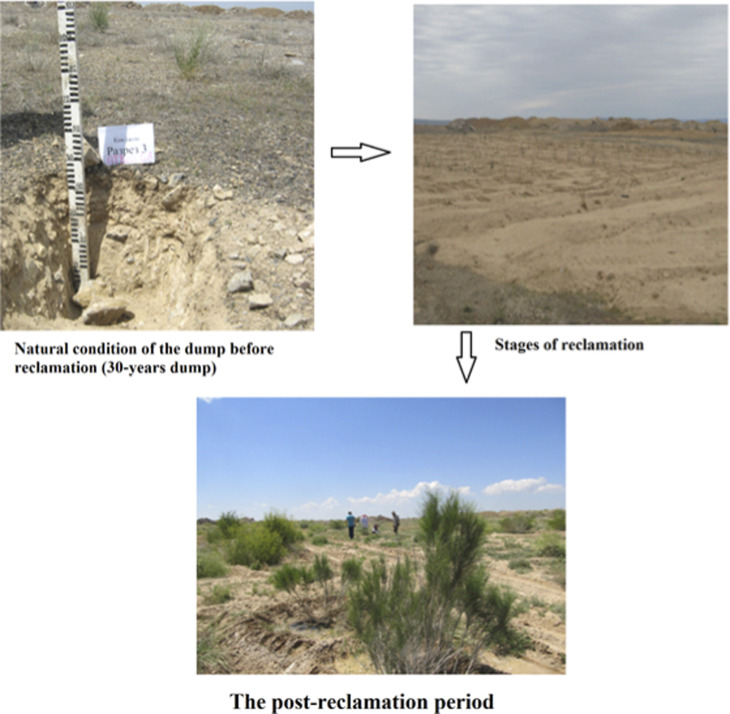
Condition of the dump before reclamation after reclamation.

By 2023, long-term monitoring showed a stable number of sprouted trees, indicating maturity in the reclamation process (Fig 6). Androsov Elm maintained 27 sprouted trees, while Salt Cedar stabilized at 22. Although Russian Olive and Black Saxaul did not show further increases in the number of sprouted trees, their height growth was significant, with Russian Olive reaching up to 220 cm and Androsov Elm achieving heights up to 230 cm. This stability in survival and continued growth highlights the sustainability of the reclamation strategy and the effectiveness of the selected tree species.

Differences in survival rates among the four species were statistically significant, as demonstrated by Fisher’s Exact Test. This analysis confirmed that Androsov Elm and Salt Cedar exhibited higher survival rates than Russian Olive, showcasing distinct species-specific adaptation capabilities. The overall survival rate of 11% was calculated with a confidence interval of 8.3% to 14.2%, highlighting variability in survival rates (ranging from 1.3% to 18%). These results underscore the importance of testing multiple species to identify the most resilient candidates for reclamation in semi-arid environments.

These findings align with Adenova et al. [39], who demonstrated the vital role of herbaceous plants in initiating soil recovery in arid and semi-arid reclamation sites. Herbaceous plants are particularly effective in restoring vegetation cover in degraded landscapes due to their resilience in adverse environmental conditions. They enhance soil structure, reduce erosion, improve moisture retention, and facilitate the self-propagation of seeds in remote areas of the reclaimed experimental site [45–47].

The results of reclamation efforts between 2011 and 2018 demonstrated significant improvements in soil quality and vegetation cover on the reclaimed dump, especially when compared to nearby regional soils unaffected by industrial activities. Regional soils, located outside the mining area, typically exhibit higher baseline fertility due to the absence of industrial disturbance and retain a natural vegetation cover of 70 - 80%. These zonal soils have historically higher humus content (1.2% in 2011) and support greater biological productivity of herbaceous plants (2400 kg/ha in 2011), as shown in Table 5.

In the context of industrial reclamation, the humus content and vegetation productivity of the reclaimed dump have approached - and in some aspects exceeded - the conditions of regional soil. By 2018, the reclaimed dump exhibited a humus content of 1.1% and a biological productivity of 3300 kg/ha for herbaceous plants, surpassing the 0.77% humus content and 2000 kg/ha productivity of regional soils during the same year. These findings align with studies by Toktar et al. [13] and Adenova et al. [39], which emphasize the critical role of soil amendments and phytomeliorants in accelerating fertility recovery in degraded soils.

By 2023, the humus content on the reclaimed dump slightly increased from 1.10% in 2018 to 1.14%, and the biological productivity of herbaceous plants reached 3350 kg/ha. In contrast, regional soils located 1 km from the dump exhibited lower humus content (0.7%) and a biological productivity of 1900 kg/ha, likely due to natural soil depletion and external environmental factors. These results reflect the resilence of reclaimed soils under managed conditions, consistent with finding by Toktar et al. [13] who highlighted the importance of ongoing soil management to sustain and enhance gains in fertility and productivity over time. These observations underscore the success of reclamation efforts in restoring soil fertility and vegetation cover on previously degraded industrial land. Moreover, the ongoing need for sustainable soil management practices to maintain and build upon these improvements ensuring the long-term functionality of reclaimed ecosystems.

## Conclusions

The structure of agricultural soil comprises approximately 50% solid material, with the remainder consisting of pores filled with water and air. Among solids, clay and organic compounds significantly influence soil quality, determining its agricultural properties. Industrial activities disrupt soil structure, often rendering it unsuitable for agricultural or ecological use. Biochar application addresses several critical challenges, including improving long-term soil fertility, recycling agricultural waste, and sequestering atmospheric carbon. Additionally, biochar offers a cost-effective and stable solution for enhancing soil quality and mitigating heavy metal pollution.

Based on the research findings, reclamation of phosphorite deposits in the desert region has facilitated the formation of new agro-landscapes and forested areas. The strategic selection of plant species aligns with the broader goal of transforming industrial waste sites into ecologically functional landscapes. By promoting vegetation cover, improving soil health, and enhancing biodiversity, these reclamation efforts aim to restore ecological balance while creating spaces that can be repurposed for public or agricultural use.

While biochar and carbamide effectively enhance soil fertility and support vegetation growth, they have limitations. The high costs and labor-intensive application of biochar highlight the need for explore cost-effective alternatives, such as compost or green manure. Combining biochar with these alternatives could enhance its efficacy by leveraging complementary mechanisms for nutrient release, organic matter accumulation, and soil structure improvement. Long-term monitoring and experimentation with different amendment combinations will be essential for optimizing reclamation strategies and addressing site-specific conditions.

Phytomeliorants are expected to play a key role in achieving long-term reclamation objectives. The reclaimed landscape performs several important ecological functions, including: 1) restoring soil fertility by enhancing biological activity, improving physical and chemical properties, enriching the soil with organic matter, and facilitating new soil formation processes. 2) supporting food security 3) improving the safety of humans and animals 4) increasing environmental quality 5) reducing carbon dioxide and emissions into the environment. 6) creating a microclimate in desert and semi-desert conditions. 7) contributing to global warming mitigation.

## Supporting information

S1 File Inclusivity-in-global-research-questionnaire. (DOCX)
